# Employing post classification comparison to detect land use cover change patterns and quantify conversions in Abakaliki LGA Nigeria from 2000 to 2022

**DOI:** 10.1038/s41598-024-59056-w

**Published:** 2024-04-24

**Authors:** Francis E. Onuegbu, Anthony U. Egbu

**Affiliations:** Department of Urban and Regional Planning, Abia State University, Uturu, Nigeria

**Keywords:** Land use change detection, Land cover change, Post-classification comparison, Urban expansion, Remote sensing, Abakaliki, Nigeria, Environmental social sciences, Solid Earth sciences

## Abstract

Rapid urbanization is restructuring landscapes across sub-Saharan Africa. This study employed post-classification comparison of multi-temporal Landsat imagery to characterize land cover changes in Abakaliki Local Government Area, Ebonyi State, Nigeria between 2000 and 2022, addressing the need for empirical baselines to guide sustainable planning. Four classes were considered and images classified with overall accuracy of 95% for the year 2000 and 97% for the year 2022. Notably, 21,000 hectares of vegetation were lost, while built-up and bare land increased by 7500 and 13,700 hectares respectively. Spatial patterns revealed built-up encroachment from vegetation and bare land; this establishes the first standardized quantification of Abakaliki LGA’s shifting landscape, with results supporting compact development models while conserving ecological services under ongoing transformations. The study makes a significant contribution by establishing an empirical baseline characterizing Nigeria's urbanization trajectory essential for evidence-based stewardship of regional resources and livelihoods in a period of accelerating change.

## Introduction

Land cover change is a global phenomenon that significantly impacts sustainable development progress. The United Nations Sustainable Development Goals (SDGs) recognize the importance of effectively managing shifts in land use to achieve critical targets related to poverty, food security, sustainable cities and communities, climate action, terrestrial ecosystems and biodiversity^1^. Understanding spatial and temporal patterns of landscape transformations provides an empirical evidence base to balance competing demands on land through coordinated policymaking and planning aligned with the integrated landscape approach (ILA) framework.

ILA is an established theoretical concept emphasizing collaborative governance across sectors to analyze landscape mosaics and interactions over time, guiding strategies that promote both human well-being and ecological resilience objectives outlined in the SDGs^2^. Applying ILA in practice necessitates quantitative data characterizing historical land change dynamics within specific local contexts. Where such information is limited, spatial planning risks being misaligned with on-the-ground realities, hindering seamless SDG implementation. Remote sensing techniques allow retrospective analysis of past and ongoing conversions across large areas and periods, overcoming data deficiencies.

Post-classification comparison (PCC) is a standardized methodology for measuring land cover changes between dates by independently classifying imagery acquired at different points and comparing results^3^. Globally, PCC has been widely adopted given its accuracy and suitability for mapping spatial patterns and quantifying conversions, in sub-Saharan Africa PCC applications have predominantly focused on regional transformations associated with rapid urbanization in megacities or transformations of rural agricultural landscapes, with limited insights available regarding mid-sized urbanizing areas^4,5^.

As secondary cities across Africa experience extensive population increases and economic growth, understanding landscape dynamics at this scale is imperative to guide sustainable compact development achieving SDG targets. Yet localized quantitative data characterizing conversions remains scarce, constraining empirical grounding of ILA-based policies balancing priorities like housing, industry, food security and biodiversity conservation as mid-urban settlements transform. Addressing this knowledge gap is a research priority.

In Nigeria, urban expansion is projected to significantly reshape landscapes amid the country's projected rise to third largest global population by 2050^6^. Studies have overwhelmingly centered on megacities, neglecting insights into smaller urbanizing settlements. Abakaliki LGA, Ebonyi State, Nigeria, is one such context of rapid urbanization and associated infrastructure growth. Between 2000 and 2022, Abakaliki LGA population surged over 300% from 57,000 to 240,000, anticipated to reach 500,000 by 2040^7,8^.

Nonetheless, quantitative characterization of landscape conversions linked to Abakaliki LGA’s development trajectory remains limited. This research gap hinders adoption of evidence-based ILA approaches to balance competing land claims as the cities expand. The current study addresses this through employing standardized multi-temporal PCC of Landsat imagery to detect and quantify land cover changes in Abakaliki Local Government Area from 2000 to 2022.

Findings will provide the first empirically grounded understanding of patterns and rates of transformations experienced over two decades of urbanization in Abakaliki LGA. This supports optimized integrated spatial planning to harmonize housing, industry, agriculture, green space provision and biodiversity stewardship objectives as per the 2030 Agenda^1^. The research also contributes to addressing the paucity of localized quantitative land change analysis in West Africa through application of advanced remote sensing techniques at the mid-urban scale.

## Materials and methods

### Study Area

**Figure 1:**
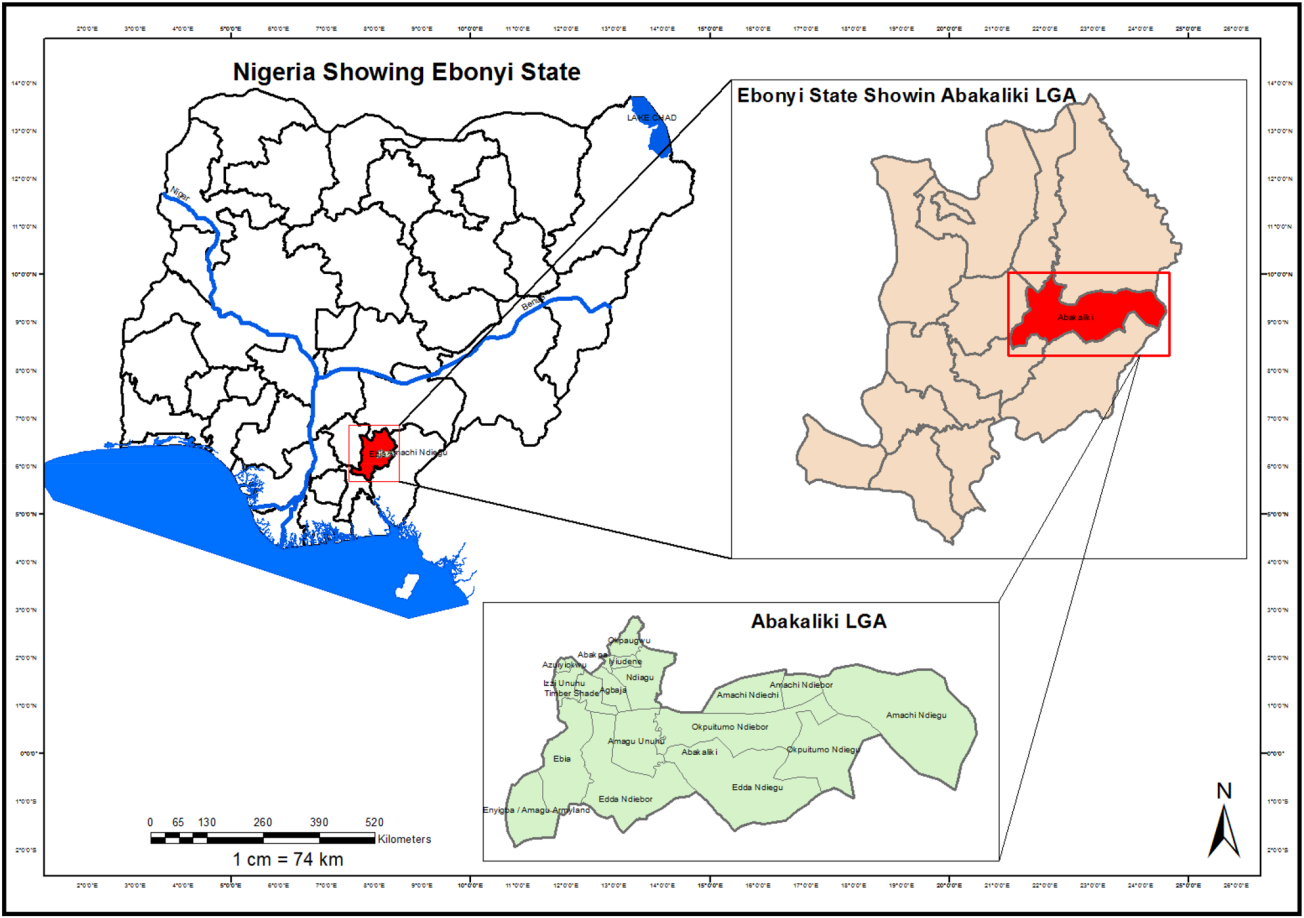
Map of the study area. Created with: ARCGIS 10.4.1 version. https://desktop.arcgis.com/en/quick-start-guides/10.4/arcgis-desktop-quick-start-guide.htm.

Abakaliki Local Government Area, Ebonyi State, Nigeria, served as the study area for investigating land cover change dynamics over two decades. Situated between Latitude 5°32’–5°42’ N and Longitude 7°58’–8°12’ E, Abakaliki LGA encompasses approximately 540 km^2^ of undulating terrain ranging from 70 to 150 m above sea level (Fig. 1). The region experiences a tropical climate characterized by a distinct wet season from April to October and drier season from November to March (NIMET, 2022). On average, annual rainfall totals 1500–2000 mm while average temperatures vary within a narrow band of 22–32 °C year-round (NIMET, 2022).

This climate supports diverse agricultural production critical to the local economy. Historically, the landscape surrounding Abakaliki consisted primarily of fragmented cultivated lands interspersed with patches of secondary tropical forest and woodland savanna^9^. In recent decades rapid population growth has driven widespread conversion of outlying areas for settlements and expansion of industrial and service sectors^10^. Between 1990 and 2015, Abakaliki LGA witnessed over 300% surge in inhabitants from 57,000 to 240,000 due to rural–urban migration and natural increase^7,8^.

**Figure 2:**
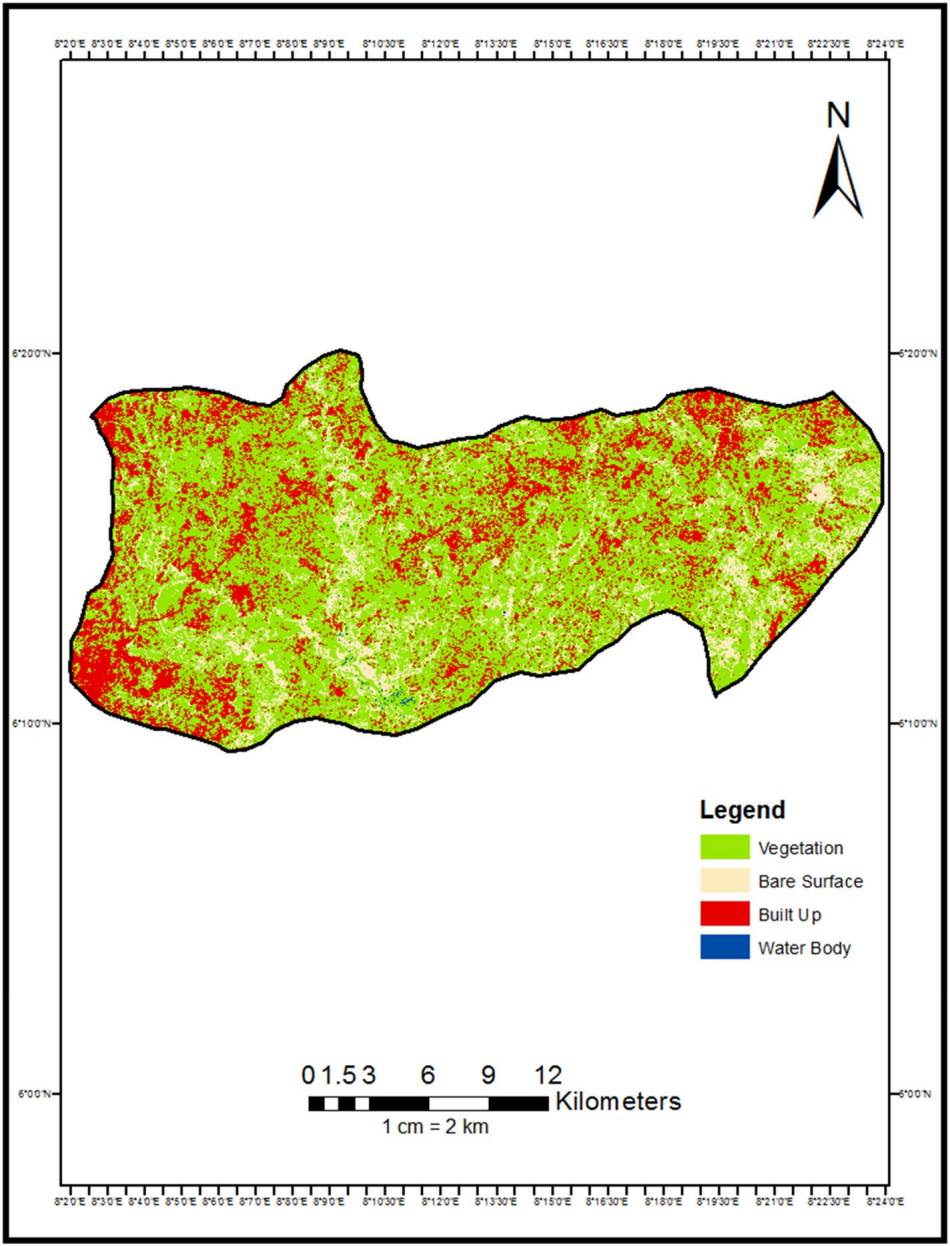
Land use land cover map of Abakaliki LGA 2000.

**Figure 3:**
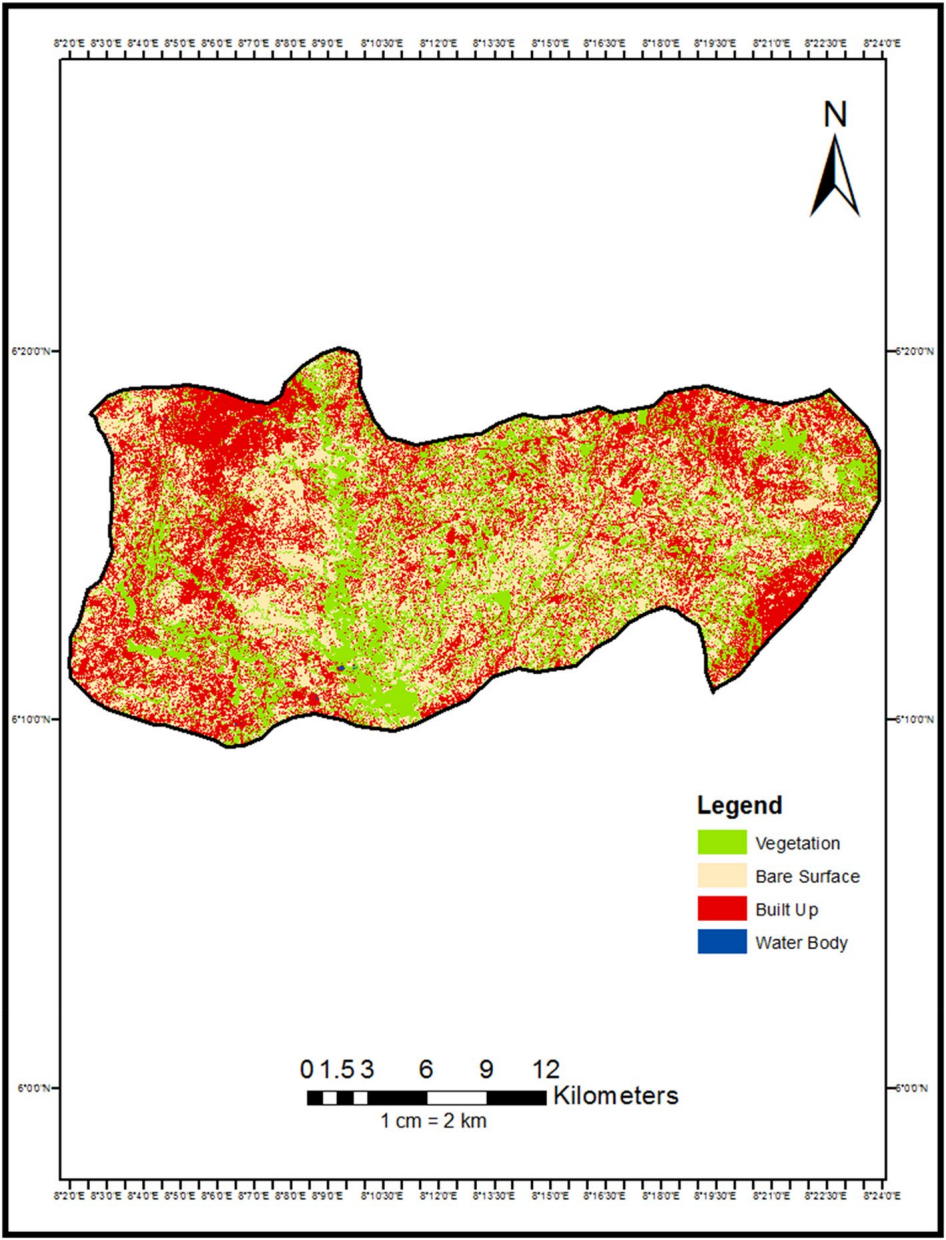
Land use land cover map of Abakaliki LGA 2022.

Despite transformations accompanying recent development, Abakaliki LGA retains its designation as Ebonyi State capital and hub for regional trade, public administration, and agricultural processing^11^. However, accelerating urbanization poses sustainability challenges if unplanned (Figs. 2 and 3).

## Materials and methods

This study employed a post-classification comparison (PCC) approach to analyze land cover changes in Abakaliki, Nigeria between 2000 and 2022. Multi-temporal Landsat 7/8 imagery from 2000 to 2022 were obtained from the United States Geological Survey (USGS) Earth Explorer^12^. Images from the dry seasons (November-March) were selected to minimize moisture variations^13^.

A hybrid object-based image analysis and pixel-based maximum likelihood classification was conducted in eCognition Developer 10.1 and ArcGIS Pro 2.8^14^. A multi-resolution segmentation algorithm segmented spectrally similar pixels into image objects at optimal scales^15^. Maximum likelihood classification is a parametric method that assigns each pixel to the class that has the highest probability or likelihood, based on the statistics derived from the training samples^16^. Objects and pixels were assigned to land cover classes—vegetation, built up, water body and bare surface sing reference data^16^.

A total of 1000 random points were generated for each land cover class present. 67% of the points (667) were used for training the classifier, while the remaining 33% (330) comprised the testing samples for the accuracy assessment. Classification accuracies were assessed through error matrices generated from validation points collected via field surveys and very high resolution Google Earth imagery^17^. PCC was performed by cross-tabulating the 2000 and 2022 classification maps to quantify area changes between classes, To assess the accuracy of the classified maps, 330 randomly selected reference points per class were identified using high resolution imagery (Tables 1, 2). The reference points were overlaid on the classified maps and compared to calculate the producer's and user's accuracies using the following equations^18^(Tables 3 and 4).

**Table 1:** Accuracy assessment for LULC Map 2000.

	Water body	Built-up	Vegetation	Bare surface	Total (user)
Water body	26	0	4	0	30
Built-up	0	95	1	3	100
Vegetation	0	0	100	0	100
Bare surface	0	7	0	93	100
Total (producer)	26	102	105	96	330

**Table 2:** Accuracy assessment for LULC Map 2022.

	Water body	Built-up	Vegetation	Bare surface	Total (user)
Water body	28	0	2	0	30
Built-up	0	98	1	1	100
Vegetation	0	0	100	0	100
Bare surface	0	5	0	95	100
Total (producer)	28	103	103	96	330

**Table 3:** Result land use land cover map of Abakaliki LGA 2000.

Class name	Sum of area in SQkm	% of land cover
Bare surface	63.459892	11.8
Built up	123.125234	23.0
Vegetation	349.02873	65.1
Water bodies	0.529529	0.1
Grand total	536.143385	100.0

**Table 4:** Result land use land cover map of Abakaliki LGA 2022.

Class name	Sum of area in SQkm	% of land cover
Bare surface	200.615782	37.42
Built up	198.481344	37.02
Vegetation	136.900275	25.54
Water bodies	0.097056	0.02
Grand total	536.143385	100.00

## Results

**Figure 4:**
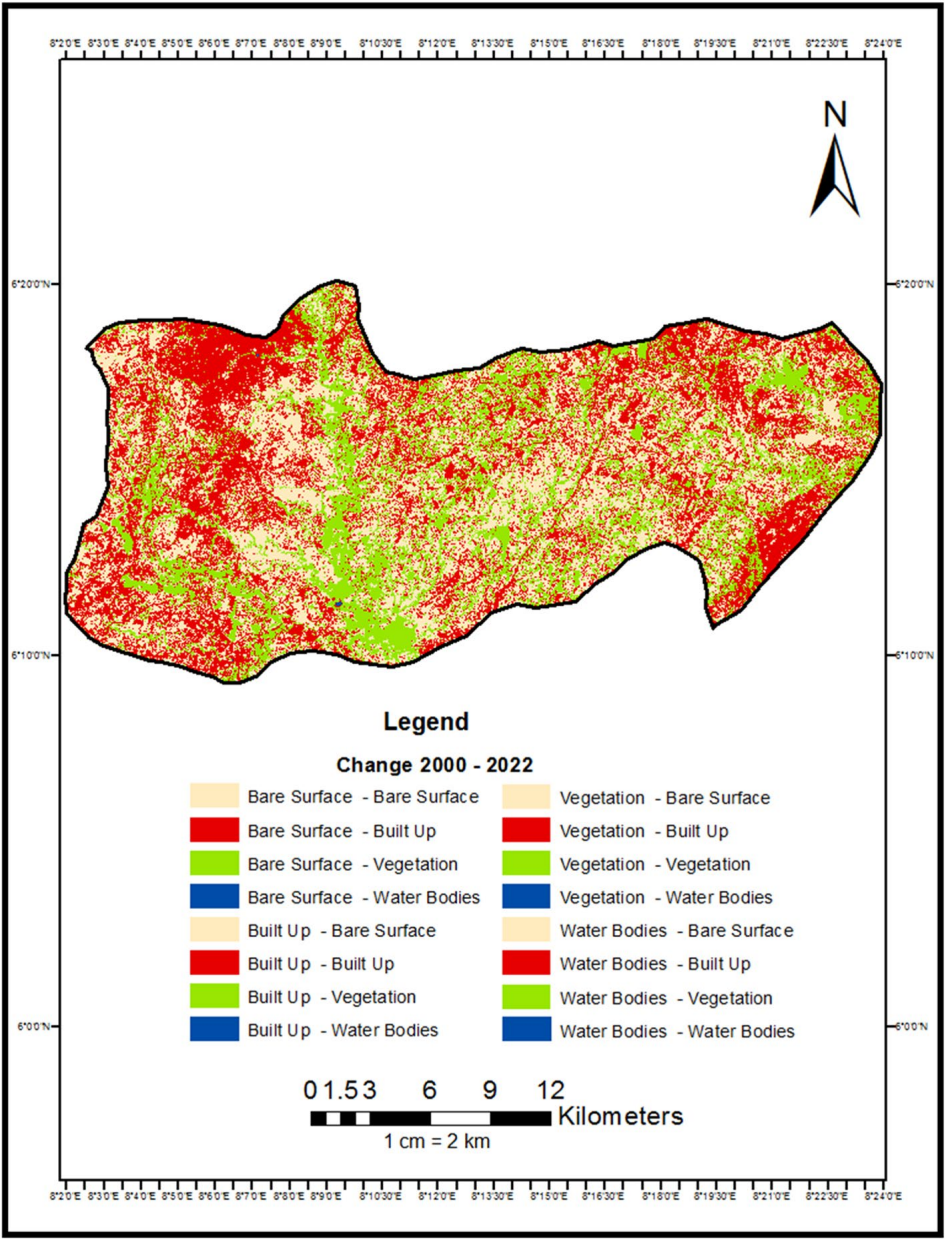
Change detection map of Abakaliki LGA 2000 to 2022.

**Figure 5:**
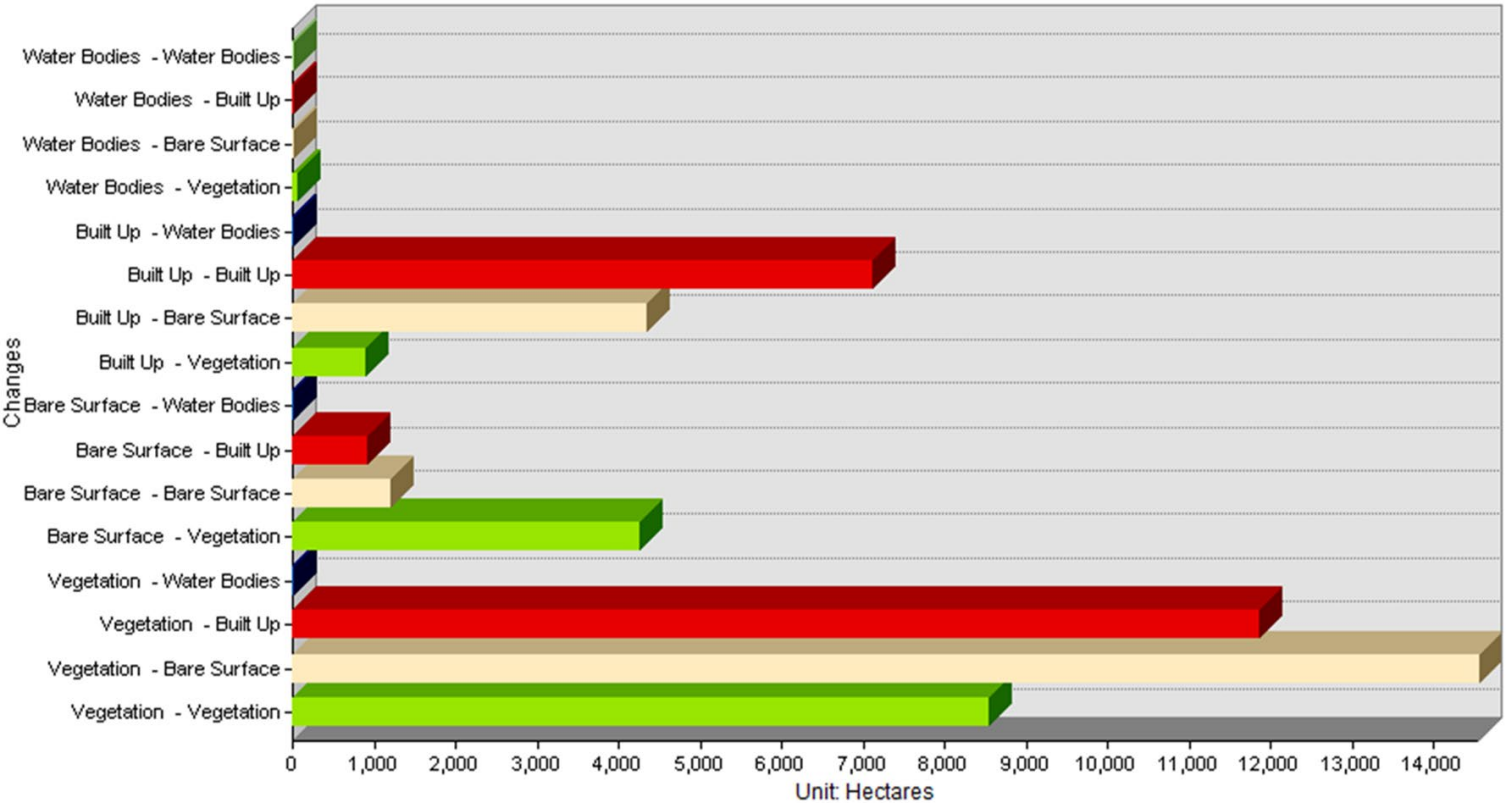
Bar chart representation of Abakaliki LGA change detection result 2000 to 2022.

The following section presents the key findings that emerged from applying the post-classification comparison approach to detect landscape changes in Abakaliki, Nigeria between 2000 and 2022. First, quantitative results from the change detection analysis are reported. Area changes between land cover classes are shown numerically in Table 5 and visually depicted in the change detection map (Fig. 4). A bar chart (Fig. 5) further illustrates conversions. Trends in overall class changes are then quantified and summarized in Table 6, Fig. 5. Having objectively documented transformations at the pixel level, spatial patterns revealed by the analysis are described. Insights into the nature, location, and magnitude of landscape conversions over the study period provide empirical insights into the trajectory of environmental changes accompanying Abakaliki's rapid urbanization.

$$ {\mathbf{Overall\, Accuracy}}  = \frac{{Total\,Number\,of\,Correctly\,Classified\,Pixels}}{{Total\,Number\,of\,Reference\,Pixels}} \times { }100 = 95\% $$

**Table 5:** Change detection result 2000 to 2022.

Change (2000 to 2022)	Area in hectares
Bare Surface–Bare Surface	1185.942599
Bare Surface–Built Up	909.91108
Bare Surface–Vegetation	4245.591269
Bare Surface–Water Bodies	2.920773
Built Up–Bare Surface	4329.896808
Built Up–Built Up	7098.698947
Built Up–Vegetation	878.228337
Built Up–Water Bodies	1.322013
Vegetation–Bare Surface	14,541.93343
Vegetation–Built Up	11,832.63013
Vegetation–Vegetation	8514.632083
Vegetation–Water Bodies	4.915607
Water Bodies–Bare Surface	0.417812
Water Bodies–Built Up	2.667922
Water Bodies–Vegetation	49.303622
Water Bodies–Water Bodies	0.547186

**Table 6:** % of Change from 2000 to 2022.

Class name	Hectares	% of change	Remark
Bare surface	13,715.59	25.59	Increase
Built up	7535.61	14.06	Increase
Vegetation	− 21,212.84	− 39.56	Decrease
Water bodies	− 43.25	− 0.08	Decrease

**User Accuracy**

$$ {\text{User\, Accuracy}} = \frac{Total\, Number\, of\, Correctly\, Classified\, Pixels\, in\, Each\, Category}{{Total\, Number\, of\, Reference\, Pixels\, in\, that\, Category}} \times 100 $$

$$ {\text{Water Body}} = \frac{26}{{30}} \times 100 = 87\% $$

$$ {\text{Built-up}} = \frac{95}{{100}} \times 100 = 95\% $$

$$ {\text{Vegetation}} = \frac{100}{{100}} \times 100 = 100\% $$

$$ {\text{Bare Surface }} = \frac{93}{{100}} \times 100 = 93\% $$

$$ {\mathbf{Producer\,Accuracy}} = \frac{Total\, Number\, of\, Correctly\, Classified\, Pixels\, in\, Each\, Category}{{Total\, Number\, of\, Reference\, Pixels\, in\, that\, Category \left( {Column\, Total} \right)}} \times 100 $$

$$ {\text{Water Body}} = \frac{26}{{26}} \times 100 = 100\% $$

$$ {\text{Built-up}} = \frac{95}{{102}} \times 100 = 93\% $$

$$ {\text{Vegetation}} = \frac{100}{{105}} \times 100 = 95\% $$

$$ {\text{Bare Surface}} = \frac{93}{{96}} \times 100 = 97\% $$

$$ {\mathbf{Kappa\, Coefficient}} \, \left( {\mathbf{T}} \right) = \frac{{\left( {TS \times TCS} \right) - \sum \left( {Column\, Total + Row \,Total} \right)}}{{TS^{2} - \sum \left( {Column\, Total + Row\, Total} \right)}} \times 100 = {\mathbf{87}}\% $$

where: TS = Total Samples; TCS = Total Correctly Classified Samples.

$$ {\text{Overall Accuracy}} = \frac{Total\, Number\, of\, Correctly\, Classified\, Pixels}{{Total\, Number\, of\, Reference\, Pixels}} \times 100 = 97\% $$

**User Accuracy**

$$ {\text{Water Body}} = \frac{28}{{30}} \times 100 = 93\% $$

$$ {\text{Built-up}} = \frac{98}{{100}} \times 100 = 98\% $$

$$ {\text{Vegetation}} = \frac{100}{{100}} \times 100 = 100\% $$

$$ {\text{Bare Surface}} = \frac{95}{{100}} \times 100 = 95\% $$

**Producer Accuracy**

$$ {\text{Water Body}} = \frac{28}{{28}} \times 100 = 100\% $$

$$ {\text{Built-up}} = \frac{98}{{103}} \times 100 = 95\% $$

$$ {\text{Vegetation}} = \frac{100}{{103}} \times 100 = 97\% $$

$$ {\text{Bare Surface}} = \frac{95}{{96}} \times 100 = 98.9\% $$

$$ \user2{Kappa\, Coefficient }\left( {\varvec{T}} \right)\user2{ } = 96\user2{\% } $$

Spatial patterns of landscape transformation were analyzed to discern anthropogenic and environmental factors reshaping Abakaliki from 2000 to 2022^19^. This PCC approach optimized land change detection at Landsat scales to detect long-term trends to inform sustainable urban planning balancing development and conservation in Abakaliki^20^.

From the change detection analysis, it shows that 1185.942599 hectares of land was converted from Bare Surface to Bare Surface, 909.91108 hectares of land was converted from Bare Surface to Built Up, 4245.591269 hectares of land was converted from Bare Surface to Vegetation, 2.920773 hectares of land were converted from Bare Surface to Water Bodies, 4329.896808 hectares of land was converted from Built Up to Bare Surface, 7098.698947 hectares of area covered by Built Up remained unchanged, 878.228337 hectares was converted from Built Up to Vegetation this could be attributed to buildings that were demolished or converted to other uses though to government policy, 1.322013 hectares of were converted from Built Up to Water Bodies, 14,541.93343 hectares of land were converted from Vegetation to Bare Surface, 11,832.63013 hectares of land was converted from Vegetation to Built Up, 8514.632083 hectares of land covered by Vegetation remains unchanged, 4.915607 hectares of Vegetative was changed to Water Body, 0.417812 hectares of land covered by water body was converted to Bare Surface, 2.667922 hectares of land covered by Water Bodies was converted to Built Up area, 49.303622 Hectares of land was converted from Water Bodies to Vegetation, while 0.547186 hectares of land covered by Water Body remained unchanged.

**Figure 6:**
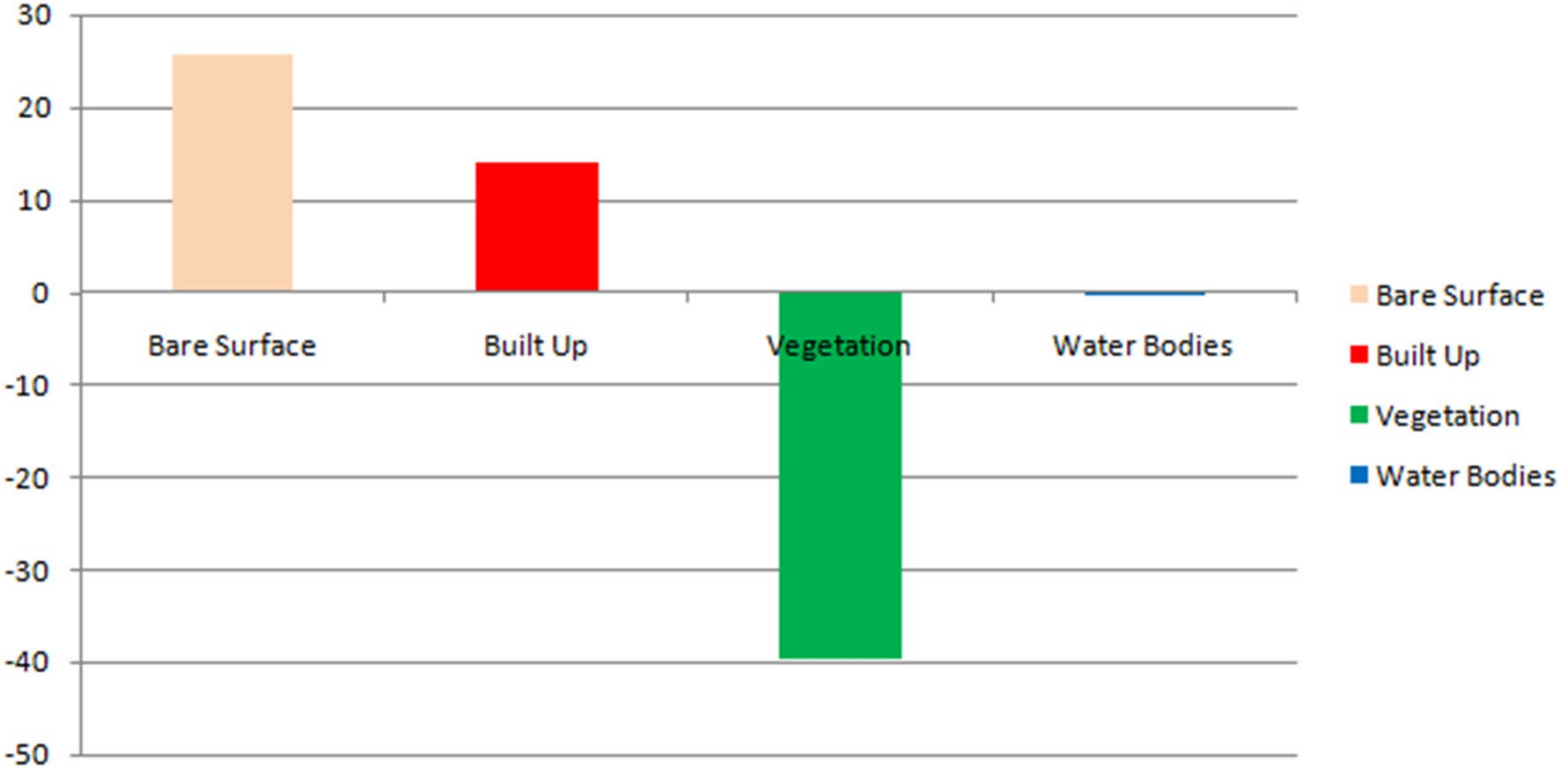
Graphical representation of % of change from 2000 to 2022.

From Table 6 and Fig. 4, it shows that from the year 2000 to 2022, bare land experience a total increase of 13,715.59 Hectares amounting to 25.59% increase, this could be attributed to deforestation and bush burning as a result of agricultural practices in the area, built up incurs an increase of 7535.61 hectares amounting to 14.06% increase in the total Land area, this can be attributed to urbanization and industrialization, vegetation incurs a decrease − 21,212.84 hectares of land amounting to − 39.56% loss in the vegetative cover, while Water bodies had a decrease of − 43.25 hectares of land area amounting to -0.08% decrease in water body (Fig. 6).

## Discussion

The results provide valuable insights into landscape changes unfolding in Abakaliki over the study period from 2000 to 2022. Several key trends are evident from the post-classification comparison.

Firstly, vegetative land experienced the most significant decline at over 21,000 hectares lost, likely reflecting conversion to agricultural and urban uses^21^. Bare land and built-up areas both increased substantially by over 13,000 and 7500 hectares respectively, coinciding with Abakaliki's rapid population growth and economic development^22^. Spatially, the change detection map (Fig. 4) illustrates the expanding urban fabric surrounding the city core, characterized by loss of woodlands and farmland patches to housing and industry^23^. Agricultural expansion onto former forested areas is also visually apparent^24^.

Notably, over 70% of original built pixels remained stable, suggesting urban infill and redevelopment rather than sprawl as a dominant dynamic^25^. However, the over 25% rise in bare land coverage may signal degradation of marginal cultivated zones^26^. The quantitative and spatial insights afforded by PCC validation that urbanization pressures chiefly drive Abakaliki's shifting landscape mosaic. Ongoing intensification requires strategic land use planning to balance growth needs without compromising long-term productivity or ecosystem services on which livelihoods rely^27^. Nonetheless, compact development trends bode well if properly managed.

This study provides a standardized empirical baseline characterizing two decades of transformation essential for evidence-based policymaking aligned with sustainability objectives as urbanization continues. Continued monitoring will track impacts and guide interventions.

## Conclusion

The aim of this study were to employ post-classification comparison of Landsat imagery to detect land cover changes in Abakaliki LGA, Nigeria from 2000 to 2022. Key findings showed vegetation decline and expansion of built-up and bare surfaces, primarily driven by urbanization. Notably, over 21,000 hectares of vegetation were lost, while built-up and bare land increased by over 7500 and 13,700 hectares respectively. Spatial patterns revealed urban encroachment onto agricultural and forested lands. This quantifies Abakaliki's shifting landscape mosaic under rapid urban development. Limitations include coarser spatial resolution and unexplored socioeconomic drivers. Continued monitoring is needed to fully characterize long-term impacts.

These results provide empirical baselines to optimize spatial planning and guide sustainable intensification efforts. Complementing remote sensing with socioeconomic analyses presents opportunities for more robust policy guidance. Prioritizing compact development models and revegetation strategies can build resilience amid intensifying pressures across Southeast Nigeria's urbanizing regions. Overall, this study establishes a critical foundation for evidence-based stewardship of Abakaliki's transforming landscape and resources through applied land change detection. Continued evaluation employing integrated approaches can further elucidate dynamics and inform adaptive solutions ensuring long-term sustainability (Supplementary information file 1).

### Supplementary Information

Supplementary Information.

## Data Availability

The Landsat imagery data that support the findings of this study were obtained from the United States Geological Survey (USGS) Earth Explorer database and are publicly available at https://earthexplorer.usgs.gov/. The processed data generated from the imagery are available from the corresponding author upon reasonable request.
