# Supplementary material for: Employing post classification comparison to detect land use cover change patterns and quantify conversions in Abakaliki LGA Nigeria from 2000 to 2022
Source: Sci Rep. 2024 Apr 24;14:9384. doi: 10.1038/s41598-024-59056-w (PMC11039723; doi:10.1038/s41598-024-59056-w)
Supplement: Supplementary file 1 — Supplementary Information. [file 41598_2024_59056_MOESM1_ESM.docx]

**Appendix**


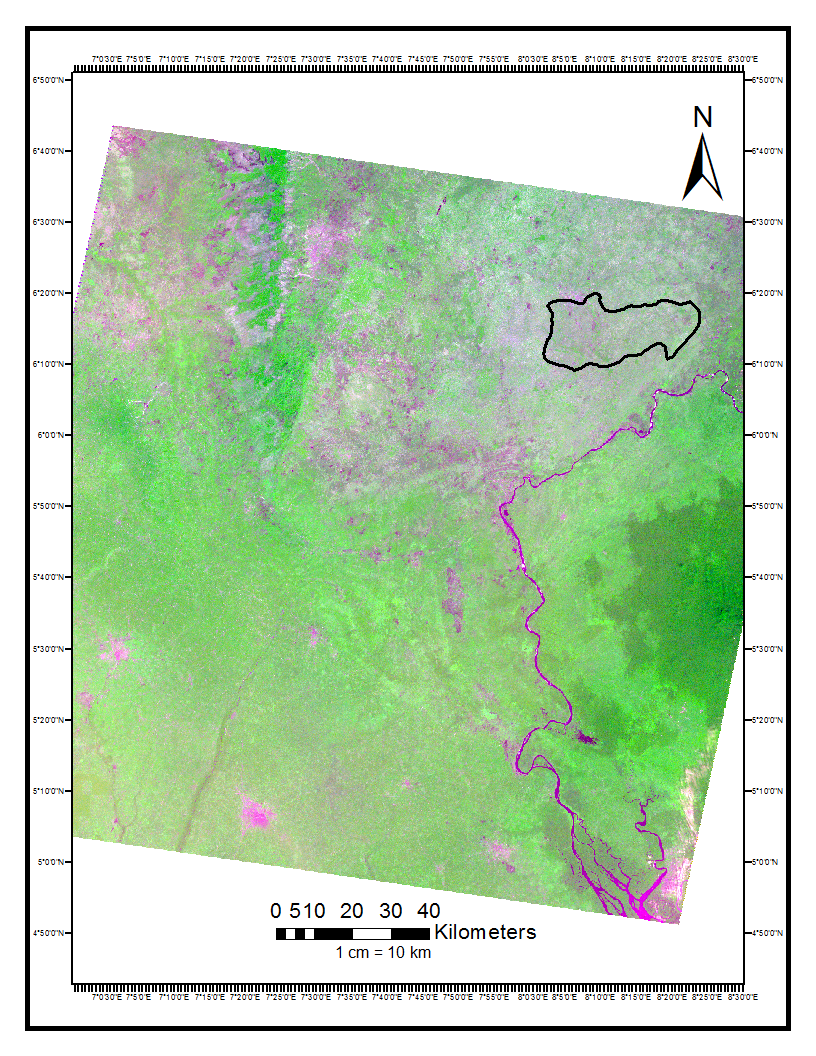


**Fig. 4: Unclassified Landsat 7 Image for the year 2000**

***Source: United State Geological Survey (USGS)***

***
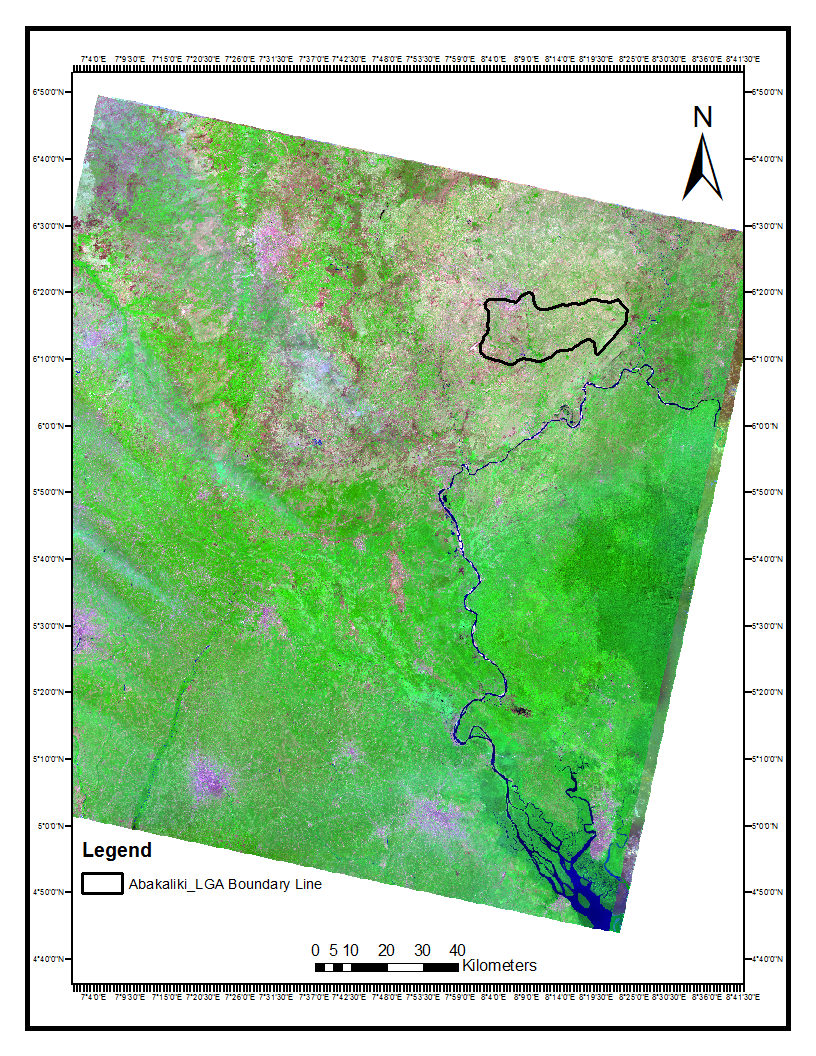
***

**Fig. 5: Unclassified Landsat 8 Image for the year 2022**

***Source: United State Geological Survey (USGS)***
